# The pattern of physical disability and determinants of activities of daily living among people with diabetes in Bangladesh

**DOI:** 10.1002/edm2.365

**Published:** 2022-08-14

**Authors:** Sabrina Ahmed, Mithila Faruque, Mohammad Moniruzzaman, Naym Uddin Roby, Fatema Ashraf, Yuichiro Yano, Katsuyuki Miura, M. S. A. Mansur Ahmed

**Affiliations:** ^1^ Department of Noncommunicable Diseases Bangladesh University of Health Sciences Dhaka Bangladesh; ^2^ NCD Epidemiology Research Center Shiga University of Medical Science Otsu Japan; ^3^ School of Rehabilitation Science McMaster University Hamilton Ontario Canada; ^4^ Department of Gynaecology and Obstetrics Shaheed Suhrawardy Medical College Dhaka Bangladesh

**Keywords:** activities of daily living, determinants, diabetes, disability

## Abstract

**Introduction:**

Diabetes mellitus itself is a known predictor of physical disability and impairment in activities of daily living (ADL); however, there are existing controversies about the factors explaining the association between diabetes and disability. Therefore, we assessed the possible determinants associated with ADL impairment among people with diabetes in Dhaka city, Bangladesh.

**Methods:**

We conducted a cross‐sectional study among 480 people with diabetes aged between 50 and 70 years, and attended a tertiary level hospital in Dhaka city. For determining the ADL impairment, we used the Katz Index Scoring (6 = no impairment; <6 = impairment). Age, sex, educational attainment, household expenditure, body mass index, the status of diabetes (controlled or uncontrolled), hypertension and medication adherence to anti‐diabetic drugs were included in the statistical models, and we defined any ADL impairment (Katz score <6) as an event. Multivariable logistic regression was performed to assess the significance of relevant factors.

**Results:**

The mean age of the participants was 59.0 (standard deviation [SD], 7.0) years. The majority of the participants (76.3%) had at least some sort of physical disability. In multivariable logistic regression analysis after adjusting for all covariates simultaneously, age (odds ratio [95% confidence interval]: 1.35 [1.20 to 1.75] per 1−SD increment), BMI (1.32 [1.08 to 1.21] per 1−SD increment), higher educational attainment (0.34 [0.09–0.90]), multi‐morbidity (2.79 [1.48–5.25]) and uncontrolled diabetes (1.35 [1.10–1.45]) were independently associated with ADL impairment.

**Conclusions:**

Physical disability was common, and ADL impairment was associated with age, educational attainment, BMI, multi‐morbidities and uncontrolled diabetes among the people with diabetes in Bangladesh.

## Graphical abstract

Diabetes mellitus itself is a known predictor of physical disability and impairment in activities of daily living (ADL); however, there are existing controversies about the factors explaining the association between diabetes and disability. This cross sectional study assessed the possible determinants associated with ADL impairment among diabetic patients from a tertiary level hospital in Dhaka city, Bangladesh. Activities of daily Living (ADL) impairment was associated with higher age, higher educational attainment (inversely), higher BMI, presence of existing comorbidities, and uncontrolled diabetes status among the diabetic patients in Bangladesh.

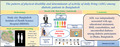

## 1 INTRODUCTION

Physical disabilities are becoming a greater burden globally^1^ and have a complex nature, including many factors like ageing, lifestyle behaviours and medical conditions.^2^ Although the improving socioeconomic status and treatment facilities have prolonged the total life expectancy, it is not the case for active life expectancy.^3^ Particularly in middle‐aged and older patients, diabetes and its complications frequently lead to various functional impairments and physical disabilities, which are observed as one of the most consistent sequelae of diabetes.^4^, ^5^ Understanding the relationship between diabetes and disability is important from several distinct perspectives. For individuals with diabetes, loss of physical functioning may be more concerning and of greater damage to quality of life.^6^ For individuals with diabetes and physicians, preventing disability is a goal, and the presence of disabilities may also affect the targets of diabetes treatment.^7^ The impact of diabetes on disability appears to be mediated through several classic diabetes complications including neuropathy, visual impairments; older age, and metabolic syndrome and is partly due to hyperglycaemia itself.^3^, ^4^, ^7^, ^8^ However, these factors do not consistently explain the association of diabetes with a disability; rather, there are inconsistencies between studies.^3^ In Bangladesh, diabetes is an alarming concern.^9^ According to the International Diabetes Federation (IDF), the cluster of low to middle‐income countries in South‐East Asia have 88 million people (aged 20–79 years) living with diabetes.^10^ In Bangladesh, the prevalence of diabetes has been increasing steadily, reaching 8.1% in 2019, and among them, 63.4% of people with diabetes have diabetes‐related complications.^10^, ^11^ As predicted by the International Diabetes Federation (IDF), the prevalence of diabetes in Bangladesh will be 13% by 2030.^10^ In Bangladesh, life expectancy at birth has increased from 47 years in 1960 to 71 years in 2015, currently 73 years. It is expected to be 78 years by 2040.^12^ The life expectancy has risen drastically, catalysed by rapid infrastructural and economic expansion in Bangladesh. With increasing life expectancy, the number of the elderly population is increasing gradually and population ageing and physical disabilities are becoming a challenge for the country.^13^ Although some Asian studies might have reported possible factors that might lead to an impairment in activities in daily living (ADL) among the people with diabetes^3^, ^14^, an existing knowledge gap remains among the Bangladeshi population. Such knowledge is crucial for both preventive and clinical views for lowering ADL impairment among individuals with diabetes. Therefore, this study aimed to assess the determinants for ADL impairment among people with diabetes in Bangladesh.

## 2 PARTICIPANTS AND METHODS

### 2.1 Study participants and setting

For this particular research, we have conducted a hospital‐based cross‐sectional study in Dhaka city, Bangladesh. In brief, the total study period was from July to November 2017. The investigators conducted a feasibility assessment for selecting a suitable healthcare centre and finally decided to conduct the study at the Bangladesh Institute of Health Science (BIHS) hospital, which is a tertiary level hospital in Dhaka especially that deals with diabetes patients. The study population was patients diagnosed with diabetes mellitus attending the selected facility for inpatient and outpatient services, both sexes and aged between 50 and 70 years. For deciding the sample size for this cross‐sectional study, we used the following formula, *n* = *z* ^2^ *p* (1–*p*)/*d* ^2^; where *n* = desired sample size. *z* = Standard error of the mean which corresponds to 95% confidence level (1.96), *p* = probability of condition being studied (0.50), and d is precision (corresponding to effect size).^15^ The initial sample size was calculated as 384. Considering the non‐response rate of 20%, we added 77 more participants, and the sample size was calculated as 461. However, we initially selected 500 participants purposively, and after excluding psychologically compromised (cannot follow instructions) *n* = 5, and severely ill individuals (*n* = 15), the remaining 480 participants with diabetes were analysed for the present study.

Written informed consent was obtained from all participants prior to the study. The study followed the code of ethics of the World Medical Association (1975 Declaration of Helsinki). The study obtained approval from the Institutional Review Board of the Bangladesh University of Health Science.

### 2.2 Measurement of ability to perform activities of daily living independently, ADL Scoring (Katz index)

The Katz Index of Independence in Activities of Daily Living, commonly referred to as the Katz ADL, is the most appropriate instrument to assess functional status as a measurement of the ability to perform ADL independently.^16^ Clinicians typically use the tool to assess function and detect problems in performing ADL and to plan care accordingly.^17^ The Index ranks adequacy of performance in the six functions of bathing, dressing, toileting, transferring, continence and feeding. Respondents scored yes/no for independence in each of the six functions. The total score ranges between 0 and 6. A score of 6 indicates full function and less than 6 indicates any impairment. The details of the Katz Index have been described in File S1.

### 2.3 Study variables

Demographic data, medical history, use of medications and lifestyle factors were collected from the participants by the trained research technicians using a structured questionnaire. Body mass index (BMI) was defined as body weight (kg) divided by the square of the height (m). Using an automated sphygmomanometer (BP‐8800SF; Omron Health Care), the mean of two consecutive measurements on the right arm with participants in a seated position after a strict 5‐minute rest period was used to determine blood pressure. Hypertension was defined as systolic blood pressure of ≥140 mm Hg, diastolic blood pressure of ≥90 mm Hg or use of antihypertensive medication.

All participants were diagnosed as having diabetes by the physicians in the facility and had a diabetes book that contains information about their current diabetes status (controlled or uncontrolled), most recent fasting blood sugar (FBS) value and HBA1c value (for the last 3 months). Fasting blood sugar was measured from venous blood after 8 h of no calorie intake, and 2 h postprandial blood sugar was estimated by measuring venous blood 2 h after meal intake. The values for FBS, 2 h of postprandial blood sugar or HbA1c were recorded in the patient's diabetes book as a routine checkup at the facility, and the interviewers included the most recent values from their diabetes book. For the current study, uncontrolled diabetes mellitus was defined as HbA1c of >6.5%. The participants were also asked about the total duration of diabetes after diagnosis and were recorded.

For identifying any physical disability, a trained physician performed a clinical visual inspection for any visible disability, such as amputation or paralysis. A visual acuity test was done by Snellen's eye chart to assess any visual difficulties. To understand the upper and lower body disability, a trained physician performed the following examinations: measuring walking speed, standing static balance, chair rise, book lift, putting on and removing a jacket, picking up a coin from the floor, turning 360 degrees, 50‐foot walk test, climbing stairs, etc.

Quality of life was measured by using the EQ‐5D‐5L Score,^18^ and medication adherence to anti‐diabetic drugs was measured using Morisky Medication Adherence Scale..^19^, ^20^ The participants were also asked about their years of education, place of residence (rural, urban and semi‐urban), occupation, monthly household expenditures and any existing chronic diseases. We have defined multi‐morbidity as the co‐occurrence of at least one chronic disease with diabetes in participants^13^ and that included the history of any sorts of diagnosed heart diseases, kidney diseases, peripheral vascular diseases, neurological insufficiencies and chronic arthritis for our study participants.

### 2.4 Statistical analysis

The participants were divided into two groups based on whether they had any ADL impairment or no impairment using the Katz Index. Participants' characteristics are shown using medians and interquartile ranges for continuous variables and percentages for categorical variables. The characteristics between the two groups were compared using the independent sample *t‐*test or Wilcoxon rank‐sum test for continuous variables and the chi‐squared test for categorical variables. We defined any ADL impairment as an event and performed a multivariable logistic regression to assess the significance with relevant factors. Age, sex, educational attainment, household expenditure, BMI, the status of diabetes (controlled or uncontrolled), hypertension and medication adherence to anti‐diabetic drugs were included in the multivariable models. Our analyses were performed with sequential adjustment: Model 1 was unadjusted, Model 2 was adjusted for age and sex, and Model 3 was simultaneously adjusted for all variables. Missing data were not included in the analyses. We also checked for the interaction of age and sex with related factors showing no persistent interaction between them (non‐significant). However, we also did an additional sex‐stratified multivariable logistic regression to assess any difference in finding for men and women groups.

All analyses were performed by SAS version 9.4 (SAS Institute, Cary, NC, USA). A two‐tailed *p*‐value of <.05 was pre‐specified to indicate statistical significance.

## 3 RESULTS

### 3.1 The proportion of physical disabilities and characteristics of the study participants

The mean age of the participants was 59.0 (standard deviation [SD], 7.0) years. Physical disabilities were common, as 76.3% (*n* = 366) of the participants had at least 1 type of physical disability. Table 1 shows among the participants 52.7%, 2.7%, 59% and 22% were having some sort of visual disabilities, amputations, lower body disabilities and paralysis of upper extremities, respectively. Regarding ADL impairment (Katz index), we found most of the participants had full function (89.5%, *n* = 430); however, 10% had any impairment. We also assessed the sorts of disability using EQ‐5D‐5L (quality of life) scoring, and we found that different grades of disabilities were common among the participants; for instance, 6.7%, 3.5%,11.9%,11.9%, and 11.3% were reported to have a severe disability in terms of mobility, self‐care, usual daily activities, pain/discomfort and anxiety, respectively. We have also assessed the proportion of the subtypes of lower body disabilities among the participants and described in Table S1.

**TABLE 1:** Proportion of different physical disabilities, activities daily living (ADL) impairment and quality of life measured by Eq‐5D‐5L variables among people with diabetes in Bangladesh (*n* = 480)

Variables	*n* (%)
Any physical disability	366.0 (76.3)
Visual Disability	252.0 (52.7)
Amputation	13.0 (2.7)
Lower body disability^a^	283.0 (59.0)
Paralysis of extremities	110.0 (22.9)
Activities of daily living (ADL), Katz Score	
Full function (score 6)	430.0 (89.5)
Any impairment (score 1–5)	50.0 (10.4)
Quality of Life (Eq‐5D‐5L)	
Mobility	
No disability (score 1)	271.0 (56.5)
Slight disability (score 2)	88.0 (18.3)
Moderate disability (score 3)	79.0 (16.5)
Sever disability (score 4)	32.0 (6.7)
Unable (score 5)	10.0 (2.1)
Self‐care	
No disability (score 1)	327.0 (68.1)
Slight disability (score 2)	88.0 (18.3)
Moderate disability (score 3)	36.0 (7.5)
Severe disability (score 4)	17.0 (3.5)
Unable (score 5)	12.0 (2.5)
Work, family and leisure activities (usual daily activities)	
No disability (score 1)	126.0 (26.3)
Slight disability (score 2)	168.0 (35.0)
Moderate disability (score 3)	121.0 (25.2)
Severe disability (score 4)	57.0 (11.9)
Unable (score 5)	8.0 (1.7)
Pain/Discomfort	
No disability (score 1)	126.0 (26.3)
Slight disability (score 2)	168.0 (35.0)
Moderate disability (score 3)	121.0 (25.2)
Severe disability (score 4)	57.0 (11.9)
Unable (score 5)	8.0 (1.7)
Anxiety/ depression	
No disability (score 1)	109.0 (22.8)
Slight disability (score 2)	174.0 (36.4)
Moderate disability (score 3)	133.0 (27.8)
Severe disability (score 4)	54.0 (11.3)
Unable (score 5)	8.0 (1.7)
Today's Health (EQ‐Vas score)	
Completely healthy (Score 100)	0.0 (0.0)
Not completely healthy (<100)	480 (100.0)

*Note*: Katz index: score 6 = full function and 1–5 = any impairment in activities daily living; quality of life (Eq‐5D‐5L Scoring): no disability = 1, slight disability = 2, moderate disability = 3, severe disability = 4, totally unable = 5; EQ‐Vas score is a component of EQ‐5D‐5L (Today's health).

^a^ Lower body disabilities were defined by limitations in walking, bathing, moving, using toilets, climbing stairs, etc. The detail has been explained in Table S1.

Table 2 shows the characteristics stratified by the presence of any ADL impairment. The group having an ADL impairment were the elderly, having a lower level of education, having lower household expenditure, higher systolic BP, having comorbidities, and also regarding EQ‐5D‐5L index, having impaired mobility, impaired self‐care, higher pain and higher anxiety compared to the no impairment group. These differences were statistically significant.

**TABLE 2:** Participants' characteristics stratified by ADL status among people with diabetes in Bangladesh (*n* = 480)

Characteristics	No Impairment (*n* = 430, 89.5%)	Impaired ADL (*n* = 50, 10.4%)	*p*‐value^a^
Age, years	58.0 (53.0–64.0)	60.0 (55.0–66.0)	.041
Sex, men	240.0 (50.0)	240.0 (50.0)	.765
Education, years, no. (%)			
No education	56.0 (13.0)	10.0 (20.0)	.027
1–5, years	83.0 (19.3)	17.0 (34.0)	
6–10, years	132.0 (30.7)	14.0 (28.0)	
11–12, years	68.0 (15.81)	4.0 (8.0)	
>12 years	91.0 (21.16)	5.0 (10.0)	
Place of residence, no. (%)			
Rural	78.0 (18.1)	14.0 (28.0)	.160
Urban	296.0 (68.8)	28.0 (56.0)	
Semi‐urban	56.0 (13.02)	8.0 (16.0)	
Household expenditure, USD	347.7 (231.6–521.2)	289.5 (231.6–347.4)	.013
Fasting blood sugar, mmol/L	8.1 (6.5–10.7)	8.8 (6.1–11.4)	.923
2 h post prandial blood sugar, mmol/L	12.0 (9.3–14.9)	13.1 (9.0–16.6)	.778
Diabetes Status, no. (%)			
Uncontrolled, (HbA1c >6.5)	281.0 (65.3)	32.0 (64.0)	.849
Diastolic BP, mmHg	80.0 (75.0–90.0)	80.0 (74.0–90.0)	.441
Systolic BP, mmHg	130.0 (120.0–140.0)	135.0 (120.0–150.0)	.043
Duration of diabetes, years	9.0 (5.0–15.0)	12.0 (7.0–17.0)	.077
BMI, kg/m^2^	26.2 (23.0–28.6)	26.2 (23.0–28.6)	.819
Waist Circumference, cm	38.0 (35.0–40.0)	38.0 (34.0–41.0)	.799
Hip Circumference, cm	39.0 (37.0–41.0)	38.0 (33.0–41.0)	.056
Family history of diabetes, yes, no. (%)	241.0 (56.0)	26.0 (52.0)	.585
Medication adherence (Morisky Scale Score), no. (%)			
Low adherence	326.0 (75.8)	33.0 (66.0)	.308
Medium adherence	82.0 (19.0)	13.0 (26.0)	
High adherence	22.0 (5.1)	4.0 (8.0)	
Hypertension, no. (%)	188.0 (43.7)	23.0 (46.0)	.758
Occupation, no. (%)			
Unemployed	104.0 (24.1)	13.0 (26.0)	.075
Service holder	48.0 (11.1)	0.0 (0.0)	
Business	11.0 (2.5)	2.0 (4.0)	
Self‐employed	59.0 (13.7)	10.0 (20.0)	
labourer	10.0 (2.3)	3.0 (6.0)	
Farmers	17.0 (3.9)	0.0 (0.0)	
House maker	181.0 (42.0)	22.0 (44.0)	
EQ‐5D‐5L scale, no. (%)			
Impaired Mobility	162.0 (37.6)	47.0 (94.0)	<.001
Impaired Self‐care	106.0 (24.6)	47.0 (94.0)	<.001
Impaired usual activities	181.0 (42.0)	22.0 (44.0)	.065
Pain/Discomfort	307.0 (71.4)	47.0 (94.0)	<.001
Anxiety	321.0 (74.6)	48.0 (96.0)	<.001
EQ‐Vas Score (health today)	65.0 (55.0–75.0)	45.0 (40.0–55.0)	<.001
Multi‐morbidity, no. (%)	119.0 (27.6)	26.0 (52.0)	<.001

*Note*: Data are expressed as median (interquartile range) for continuous variables or number (percentages) for categorical variables. The number of observations across the categories may not add up to the total given number because of missing data. Uncontrolled diabetes mellitus was defined as haemoglobin A1c of ≥6.5%. Hypertension was defined as systolic blood pressure of ≥140 mm Hg, diastolic blood pressure of ≥90 mm Hg or use of antihypertensive medication. Medication adherence was measured by using Morisky Scale (ref), and the quality of life was measured by EQ‐5D‐5L. Multi‐morbidity was defined as the co‐occurrence of at least one chronic disease along with diabetes in a participant.

^a^ Based on the independent sample t‐test for continuous variables with normal distribution/Wilcoxon rank‐sum test for continuous variables with skewed distribution and the chi‐squared test for categorical variables.

### 3.2 Determinants of ADL

The results from multivariable logistic regression are shown in Table 3. In Model 1, (unadjusted) showed that age (odds ratio [OR] per 1−SD increment, 1.33; 95% confidence interval [CI], 1.00–1.75; *p* = .043) and multi‐morbidities (OR, 2.83; 95% CI, 1.56–5.12; *p* < .001) were positively associated with ADL impairment, while household expenditure (OR per 1−SD increment, 0.30; 95% CI, 0.10–0.54; *p* = .013) and education more than 12 years (OR, 0.30; 95% CI, 0.10–0.94; *p* = .039) were inversely associated with the ADL impairment.

**TABLE 3:** Multivariable logistic regression to assess the determinants for ADL impairment among people with diabetes in Bangladesh (*n* = 480)

Variables	Model 1		Model 2		Model 3	
	OR (95% CI)	*p*‐value	OR (95% CI)	*p*‐value	OR (95% CI)	*p*‐value
Age^a^ (per 1−SD)	1.33 (1.00–1.75)	.043	1.33 (1.00–1.77)	.045	1.35 (1.20–1.75)	.046
Sex (men)	1.09 (0.60–1.96)	.765	0.96 (0.52–1.76)	.905	1.32 (0.69–2.55)	.394
Education (years)						
1–5	1.14 (0.49–2.68)	.752	1.11 (0.47–2.64)	.799	1.32 (0.53–3.72)	.549
6–10	0.59 (0.24–1.41)	.240	0.59 (0.24–1.43)	.248	0.70 (0.27–1.82)	.470
11–12	0.32 (0.09–1.10)	.072	0.33 (0.09–1.14)	.080	0.47 (0.12–1.75)	.264
>12	0.30 (0.10–0.94)	.039	0.27 (0.08–0.87)	.029	0.34 (0.09–0.90)	.043
BMI^a^ (per 1−SD)	1.06 (0.92–1.21)	.818	1.19 (1.02–1.39)	.027	1.32 (1.08–1.21)	.030
Duration of diabetes	1.03 (0.99–1.10)	.079	1.02 (0.98–1.07)	.232	1.04 (0.99–1.09)	.073
Multi‐morbidity	2.83 (1.56–5.12)	<.001	2.82 (1.54–5.14)	<.001	2.79 (1.48–5.25)	<.001
Household expenditure^a^ (per 1−SD)	0.30 (0.10–0.54)	.013	1.14 (0.93–1.41)	.190	1.45 (0.10–1.09)	.097
HbA1c (uncontrolled)	0.93 (0.51–1.73)	.849	1.10 (1.05–1.11)	.046	1.35 (1.10–1.45)	.035
Hypertension (Yes)	1.09 (0.60–1.97)	.758	1.05 (0.58–1.90)	.862	0.87 (0.46–1.64)	.676
Medication adherence to diabetic drugs						
Moderate vs. low	1.56 (0.78–3.11)	.199	1.51 (0.75–3.01)	.242	0.73 (0.21–2.43)	.608
High vs. low	1.79 (0.58–5.52)	.307	1.88 (0.60–5.82)	.271	0.84 (0.23–3.10)	.802

*Note*: Model 1 is unadjusted, Model 2 adjusted for age and sex; Model 3 includes all variables simultaneously.

Abbreviations: BMI, body mass index; SD, standard deviation. Hypertension was defined as systolic blood pressure of ≥140 mm Hg, diastolic blood pressure of ≥90 mm Hg, or use of antihypertensive medication, uncontrolled diabetes was defined as HBA1c > 6.5%. Multi‐morbidity was defined as the co‐occurrence of at least one chronic disease along with diabetes in a participant. Medication adherence for anti‐diabetic drugs was calculated by using Morisky Scale.

^a^ Data are odds ratio per 1−SD increment for continuous variables or compared with the reference group for categorical variables. Age (per 1−SD) = 7.07 years; BMI (per 1−SD) = 3.53; Household expenditure (per 1−SD) = 272.83 USD;

After adjusting by age and sex (Model 2), age (adjusted by sex, OR per 1−SD increment, 1.33; 95% CI, 1.00–1.77; *p* = .045), BMI (OR per 1‐SD increment, 1.19; 95% CI, 1.02–1.39; *p* = .027), multi‐morbidities (OR, 2.82; 95% CI, 1.54–5.14; *p* < .001) and uncontrolled HbA1c (OR, 1.10; 95% CI, 1.05–1.11; *p* = .046) were positively associated with the ADL impairment, whereas education more than 12 years (OR, 0.27; 95% CI, 0.08–0.87; *p* = .029) was inversely associated with the ADL impairment.

In the final model after adjusting will all covariates simultaneously, we found age (OR per 1−SD increment, 1.35; 95% CI, 1.20–1.75; *p* = .046), BMI (OR per 1−SD increment, 1.32; 95% CI, 1.08–1.21; *p* = .030), multi‐morbidities (OR, 2.79; 95% CI, 1.48–5.25; *p* < .001) and uncontrolled HbA1c (OR, 1.35; 95% CI, 1.10–1.45; *p* = .035) were positively associated with the ADL impairment, whereas education more than 12 years (OR, 0.34; 95% CI, 0.09–0.90; *p* = .043) was inversely associated with the ADL impairment.

Regarding the subgroup analysis in Table 4, we found among men, age (OR per 1−SD increment, 1.34; 95% CI, 1.05–1.75; *p* = .044) and multi‐morbidities (OR, 4.41; 95% CI, 1.79–10.87; *p* < .001) were positively associated with the ADL impairment, whereas education more than 12 years (OR, 0.35; 95% CI, 0.10–0.87; *p* = .356) was inversely associated with the ADL impairment. However, in terms of women, age (OR per 1−SD increment, 2.13; 95% CI, 1.26–3.59; *p* < .001), BMI (OR per 1−SD increment, 1.32; 95% CI, 1.08–1.21; *p* = .030), multi‐morbidities (OR, 2.06; 95% CI, 1.50–5.25; *p* < .001), duration of diabetes (OR, 1.07; 95% CI, 1.01–1.15; *p* = .037) and uncontrolled HbA1c (OR, 1.35; 95% CI, 1.10–1.55; *p* = .037) were positively associated with the ADL impairment.

**TABLE 4:** Subgroup analysis.

Variables	Men (*n* = 240, 50.0%)		Women (*n* = 240, 50.0%)	
	OR (95% CI)	*p*‐value	OR (95% CI)	*p*‐value
Age^a^ (per 1−SD)	1.34 (1.05–1.75)	.044	2.13 (1.26–3.59)	<.001
Education (years)				
1–5	0.92 (0.18–4.51)	.547	3.13 (0.79–12.37)	.935
6–10	0.64 (0.14–2.86)	.856	0.86 (0.21–3.54)	.960
11–12	0.49 (0.06–3.69)	.620	0.86 (0.12–5.81)	.956
>12	0.35 (0.10–0.87)	.034	0.34 (0.09–1.67)	.953
BMI^a^ (per 1−SD)	0.93 (0.94–1.09)	.356	1.32 (1.08–1.21)	.030
Duration of diabetes	1.01 (0.94–1.09)	.617	1.07 (1.01–1.15)	.036
Multi‐morbidity	4.41 (1.79–10.87)	<.001	2.06 (1.50–5.25)	<.001
Household expenditure^a^ (per 1−SD)	1.14 (0.93–1.41)	.190	1.45 (0.10–1.09)	.060
HbA1c (uncontrolled)	1.57 (0.54–4.53)	.398	1.35 (1.10–1.55)	.037
Hypertension (Yes)	1.49 (0.59–3.71)	.391	0.55 (0.19–1.54)	.259
Medication adherence to diabetic drugs				
Moderate vs. low	2.24 (0.75–6.65)	.530	1.06 (0.29–3.37)	.872
High vs. low	2.37 (0.51–10.93)	.541	0.77 (0.07–8.23)	.834

*Note*: Multivariable logistic regression to assess the determinants for ADL impairment among people with diabetes stratified by sex in Bangladesh (n = 480). The model includes all variables simultaneously.

^a^ Data are odds ratio per 1−SD increment for continuous variables or compared with the reference group for categorical variables. BMI, body mass index; SD, standard deviation. Hypertension was defined as systolic blood pressure of ≥140 mm Hg, diastolic blood pressure of ≥90 mm Hg, or use of antihypertensive medication, uncontrolled diabetes was defined as HBA1c > 6.5%. Multi‐morbidity was defined as the co‐occurrence of at least one chronic disease along with diabetes in a participant. Medication adherence for anti‐diabetic drugs was calculated by using Morisky Scale.

## 4 DISCUSSION

In this hospital‐based study among people with diabetes, ADL impairment was associated with higher age, higher BMI, presence of comorbidities and uncontrolled diabetes whereas higher educational attainment had shown a protective association. To our best knowledge, this is the first‐ever study in Bangladesh to examine the burden of physical disabilities and determinants of ADL impairment among people with diabetes in Bangladesh. Our findings are also comparable with those reported in Western and other Asian countries with similar interests.^3^, ^21^ In the current study, we estimated that 73.6% of all participants had at least one type of physical disability; similar findings were reported in the earlier studies.^3^, ^21^, ^22^ The proportion of the people with diabetes in this study that reported having an ADL impairment (10.4%) was less than The Irish longitudinal study on ageing (TILDA) (13%), the US Health and Retirement Study (18%).^23^, ^24^ The possible cause could be the current study included patients aged between 50 and 70 years, who were relatively younger (mean age 59 years) than both of these studies; for the TILDA Study (age range was >65 years to 80) and for the US Health and Retirement Study, the overall mean age was 74.6 years.

We have reported age as an independent determinant for ADL impairment similar to prior studies. A study, conducted among the elderly Japanese patient population (Japanese Elderly Diabetes Intervention Trial), has identified age as an independent factor for the new onset of ADL‐related disabilities among participants with diabetes.^3^ Furthermore, ageing has been declared as a crucial independent risk factor for ADL impairment in Western studies as well.^23^, ^25^ With the global trend of ageing, it would be expected that the burden of ADL disabilities will be increasing; however, interestingly, some studies have identified that the onset of disability can be a reversible event or can reduce over time during the ageing process.^24^, ^26^

Weight and BMI are commonly reported to play an important role in disability in ADL in the elderly population.^27^ Previous study has reported that controlling separately for BMI can reduce diabetes‐related odds of disability by 24%.^28^ The possible pathophysiology could be a lack of physical activities leads to weight gain and increased chances of metabolic syndrome leading to disability.^3^, ^29^ It has been reported that those in the normal BMI group were more engaged in leisure exercises, which might have beneficial effects on activities in daily living (*p* < .01).^29^ In our study, we have reported a positive association between BMI and ADL impairment. The assessment of ADL and BMI has yet to be a compulsory or consistent routine protocol in diabetes management practice to understand the association well; therefore, future studies are recommended.

An excess risk of disability among patients with diabetes is not surprising given the associated multi‐morbidities.^28^, ^30^ As comorbidities are prevalent,^24^ interventional programs promoting effective chronic disease management are indicated to promote active participation and to help delay the onset of ADL disabilities.^31^ Earlier studies among the US population reported that only controlling for existing comorbidities along with BMI can reduce the diabetes‐related odds by 52%.^28^ Apart from diabetes, multi‐morbidities are closely associated with ADL and instrumental activities of daily living (IADL) impairment among patients with other chronic diseases as earlier studies have revealed.^32^ However, some prior studies generated evidence that the blood glucose levels, insulin sensitivity and diabetic vascular complications were not directly linked to the development of new ADL disability; therefore, further investigations are required.^33^, ^34^

The degree to which hyperglycaemia itself explains the association between diabetes and disability remains unclear. We have found a positive association between uncontrolled Hba1c (>6.5%) and ADL impairment. An earlier study found that glycosylated haemoglobin (A1C) levels <5.5%, those with HbA1c levels of ≥8.0% had about three times the greater incidence of disabilities, adjusted for BMI and comorbidities. However, HbA1c levels in the 6.0%–7.9% range had only modest, non‐significant differences in disability incidence compared to those with lower A1c levels in women.^35^ In another cross‐sectional study, higher HbA1c levels are associated with greater walking difficulties;^36^ however, future prospective observational studies are recommended.

We found that higher education attainment has a protective effect on ADL impairment. Likewise, previous studies showed that educated elderly people had better health awareness, which was significantly associated with better ADL and IADL (instrumental activities in daily living).^37^, ^38^ A possible reason is that the educated individual may be eligible for useful social support and seek adequate medical care compared to an illiterate individual.^37^

In a subgroup analysis, we found the duration of diabetes as an additional determinant in the case of women. In an earlier study, the adjusted odds of having a physical disability including ADL impairment were higher with a longer duration of diabetes in women, compared to men and women, with insulin therapy, reported higher odds of disability [3.29 (1.94–5.58)] compared to men [2.89 (1.63–5.10)] when controlled for age, ethnicity, education and BMI.^28^ In the current study, we did not discuss the type of therapy due to a lack of particular data; however, the underlying pathophysiology is still unclear regarding the association between ADL impairment and duration of diabetes among women only. Although we have reported a positive association between multi‐morbidity and ADL impairment both in men and women with diabetes, prior studies have reported that vascular complications and the other comorbidities of diabetes might impair the activities of living in men, whereas women might be more resistant to such events.^2^, ^39^, ^40^

Several limitations of the present study warrant consideration. First, we studied only participants that obtained from a tertiary level specialized hospital where special management of health is offered and also from a single area in Dhaka, Bangladesh, which may limit the generalizability of the present results. Second, owing to the cross‐sectional study design, the true relationship between ADL impairment and its determinants might have been somewhat diluted. Third, these data lack particular variables like physical activities, smoking and drinking; therefore, we could not introduce those data in our statistical models. Finally, although the Katz ADL Index is sensitive to changes in declining health status, it is limited in its ability to measure small increments of change seen in the rehabilitation of older adults and might not assess more advanced activities of daily living. However, we believe this study has generated new knowledge regarding the determinants of ADL impairment in the South Asia context, and to our best knowledge, it would be the study that has first assessed possible determinants of ADL impairment among people with diabetes in Bangladesh.

## 5 CONCLUSION

In a group of people with diabetes in Bangladesh, the majority of the participants had at least 1 type of physical disability. Age, BMI, multi‐morbidity, uncontrolled HbA1c and educational attainment were identified as independent determinants for ADL impairment among people with diabetes. This study reported on the modifiable determinants for the activities of daily living among individuals with diabetes and underlines the importance of a healthy lifestyle including weight loss with physical activities, particularly promising approaches to reduce diabetes‐related disability, especially among the Bangladeshi Population. However, further studies are recommended to determine the impact of preventive care and diabetes management practices on disability risk.

## AUTHOR CONTRIBUTIONS

**Yuichiro Yano:** Methodology (supporting); supervision (supporting); writing – review and editing (supporting). **Sabrina Ahmed:** Conceptualization (lead); data curation (lead); formal analysis (lead); investigation (lead); methodology (lead); project administration (lead); visualization (lead); writing – original draft (lead). **M. S. A. Mansur Ahmed:** Conceptualization (lead); data curation (supporting); investigation (supporting); methodology (supporting); project administration (lead); supervision (lead); writing – review and editing (supporting). **Mohammad Moniruzzaman:** Conceptualization (supporting); methodology (supporting); writing – review and editing (supporting). **Katsuyuki Miura:** Conceptualization (supporting); methodology (supporting); supervision (supporting); writing – review and editing (supporting). **Mithila Faruque:** Conceptualization (supporting); formal analysis (supporting); methodology (supporting); supervision (supporting); writing – review and editing (supporting). **Naym Uddin Roby:** Data curation (equal); investigation (supporting); project administration (supporting); writing – review and editing (supporting). **Fatema Ashraf:** Investigation (supporting); methodology (supporting); supervision (supporting); writing – review and editing (supporting).

## CONFLICT OF INTEREST

The authors have no conflicts of interest to declare.

## Supporting information


Appendix S1

## Data Availability

The data of deidentified patients are not publicly available and will not be shared due to privacy or ethical restrictions.

## References

[1] Murray CJ , Lopez AD . Global mortality, disability, and the contribution of risk factors: Global Burden of Disease Study. Lancet. 1997;349(9063):1436‐1442. 9164317 10.1016/S0140-6736(96)07495-8

[2] Tas U , Verhagen AP , Bierma‐Zeinstra SM , Odding E , Koes BW . Prognostic factors of disability in older people: a systematic review. Br J Gen Pract. 2007;57(537):319‐323. 17394736 PMC2043327

[3] Sakurai T , Iimuro S , Sakamaki K , et al. Risk factors for a 6‐year decline in physical disability and functional limitations among elderly people with type 2 diabetes in the Japanese Elderly Diabetes Intervention Trial. Geriatr Gerontol Int. 2012;12(Suppl 1):117‐126. 22435947 10.1111/j.1447-0594.2011.00819.x

[4] Boyle JP , Thompson TJ , Gregg EW , Barker LE , Williamson DF . Projection of the year 2050 burden of diabetes in the US adult population: dynamic modeling of incidence, mortality, and prediabetes prevalence. Popul Health Metr. 2010;8:29. 20969750 10.1186/1478-7954-8-29 PMC2984379

[5] Gregg EW , Cheng YJ , Saydah S , et al. Trends in death rates among U.S. adults with and without diabetes between 1997 and 2006: findings from the National Health Interview Survey. Diabetes Care. 2012;35(6):1252‐1257. 22619288 10.2337/dc11-1162 PMC3357247

[6] Phelan EA , Anderson LA , LaCroix AZ , et al. Older adults' views of "successful aging"‐‐how do they compare with researchers' definitions? J Am Geriatr Soc. 2004;52(2):211‐216. 14728629 10.1111/j.1532-5415.2004.52056.x

[7] Lee SJ , Lindquist K , Segal MR , Covinsky KE . Development and validation of a prognostic index for 4‐year mortality in older adults. JAMA. 2006;295(7):801‐808. 16478903 10.1001/jama.295.7.801

[8] Guralnik JM , Fried LP , Salive ME . Disability as a public health outcome in the aging population. Annu Rev Public Health. 1996;17:25‐46. 8724214 10.1146/annurev.pu.17.050196.000325

[9] Saquib N , Saquib J , Ahmed T , Khanam MA , Cullen MR . Cardiovascular diseases and type 2 diabetes in Bangladesh: a systematic review and meta‐analysis of studies between 1995 and 2010. BMC Public Health. 2012;12:434. 22694854 10.1186/1471-2458-12-434 PMC3487781

[10] Williams RCS , Almutairi R , et al. IDF Diabetes Atlas. 9th ed. International Diabetes Federation; 2019. Available at: https://diabetesatlas.org/atlas/ninth‐edition/

[11] Ramachandran A , Ma RC , Snehalatha C . Diabetes in Asia. Lancet. 2010;375(9712):408‐418. 19875164 10.1016/S0140-6736(09)60937-5

[12] Bangladesh Bureau of Statistics . Bangladesh Population and Housing Census 2011. Government of the People's Republic of Bangladesh; 2015.

[13] Sara HH , Chowdhury MAB , Haque MA . Multimorbidity among elderly in Bangladesh. Aging Med. 2018;1(3):267‐275. 10.1002/agm2.12047 PMC6880734 31942503

[14] Hong X , Chen X , Chu J , et al. Multiple diabetic complications, as well as impaired physical and mental function, are associated with declining balance function in older persons with diabetes mellitus. Clin Interv Aging. 2017;12:189‐195. 28182146 10.2147/CIA.S123985 PMC5279842

[15] Pourhoseingholi MA , Vahedi M , Rahimzadeh M . Sample size calculation in medical studies. Gastroenterol Hepatol Bed Bench. 2013;6(1):14‐17. 24834239 PMC4017493

[16] Dowd S , Davidhizar R . Opening up to the Katz Index. Elder Care. 1999;11(1):9‐12. 10542502

[17] Hartigan I . A comparative review of the Katz ADL and the Barthel Index in assessing the activities of daily living of older people. Int J Older People Nurs. 2007;2(3):204‐212. 20925877 10.1111/j.1748-3743.2007.00074.x

[18] Thompson AJ , Turner AJ . A Comparison of the EQ‐5D‐3L and EQ‐5D‐5L. Pharmacoeconomics. 2020;38(6):575‐591. 32180162 10.1007/s40273-020-00893-8

[19] Martinez‐Perez P , Orozco‐Beltrán D , Pomares‐Gomez F , et al. Validation and psychometric properties of the 8‐item Morisky Medication Adherence Scale (MMAS‐8) in type 2 diabetes patients in Spain. Aten Primaria. 2021;53(2):101942. 33508739 10.1016/j.aprim.2020.09.007 PMC7844132

[20] Morisky DE , Ang A , Krousel‐Wood M , Ward HJ . Predictive validity of a medication adherence measure in an outpatient setting. J Clin Hypertens (Greenwich). 2008;10(5):348‐354. 18453793 10.1111/j.1751-7176.2008.07572.x PMC2562622

[21] Fong JH . Disability incidence and functional decline among older adults with major chronic diseases. BMC Geriatr. 2019;19(1):323. 31752701 10.1186/s12877-019-1348-z PMC6873710

[22] Kalyani RR , Saudek CD , Brancati FL , Selvin E . Association of diabetes, comorbidities, and A1C with functional disability in older adults: results from the National Health and Nutrition Examination Survey (NHANES), 1999–2006. Diabetes Care. 2010;33(5):1055‐1060. 20185736 10.2337/dc09-1597 PMC2858174

[23] Connolly D , Garvey J , McKee G . Factors associated with ADL/IADL disability in community dwelling older adults in the Irish longitudinal study on ageing (TILDA). Disabil Rehabil. 2017;39(8):809‐816. 27045728 10.3109/09638288.2016.1161848

[24] Hung WW , Ross JS , Boockvar KS , Siu AL . Recent trends in chronic disease, impairment and disability among older adults in the United States. BMC Geriatr. 2011;11:47. 21851629 10.1186/1471-2318-11-47 PMC3170191

[25] Stuck AE , Walthert JM , Nikolaus T , Büla CJ , Hohmann C , Beck JC . Risk factors for functional status decline in community‐living elderly people: a systematic literature review. Soc Sci Med. 1999;48(4):445‐469. 10075171 10.1016/s0277-9536(98)00370-0

[26] Lin SF , Beck AN , Finch BK , Hummer RA , Master RK . Trends in US older adult disability: exploring age, period, and cohort effects. Am J Public Health. 2012;102(11):2157‐2163. 22994192 10.2105/AJPH.2011.300602 PMC3471673

[27] Backholer K , Wong E , Freak‐Poli R , Walls HL , Peeters A . Increasing body weight and risk of limitations in activities of daily living: a systematic review and meta‐analysis. Obes Rev. 2012;13(5):456‐468. 22212629 10.1111/j.1467-789X.2011.00970.x

[28] Gregg EW , Beckles GL , Williamson DF , et al. Diabetes and physical disability among older U.S. adults. Diabetes Care. 2000;23(9):1272‐1277. 10977018 10.2337/diacare.23.9.1272

[29] Nwose EU , Ekotogbo B , Ogbolu CN , Mogbusiaghan M , Agofure O , Igumbor EO . Evaluation of ADL and BMI in the management of diabetes mellitus at secondary and tertiary health facilities. Diabetes Metab Syndr. 2019;13(3):2266‐2271. 31235167 10.1016/j.dsx.2019.05.033

[30] Leung V , Wroblewski K , Schumm LP , Huisingh‐Scheetz M , Huang ES . Reexamining the classification of older adults with diabetes by comorbidities and exploring relationships with frailty, disability, and 5‐year mortality. J Gerontol A Biol Sci Med Sci. 2021;76(11):2071‐2079. 34003280 10.1093/gerona/glab141 PMC8514058

[31] Connolly D , O'Toole L , Redmond P , Smith SM . Managing fatigue in patients with chronic conditions in primary care. Fam Pract. 2013;30(2):123‐124. 23520365 10.1093/fampra/cmt005

[32] Marengoni A , Akugizibwe R , Vetrano DL , et al. Patterns of multimorbidity and risk of disability in community‐dwelling older persons. Aging Clin Exp Res. 2021;33(2):457‐462. 33580869 10.1007/s40520-020-01773-z PMC7914228

[33] Bruce DG , Davis WA , Davis TM . Longitudinal predictors of reduced mobility and physical disability in patients with type 2 diabetes: the Fremantle Diabetes Study. Diabetes Care. 2005;28(10):2441‐2447. 16186277 10.2337/diacare.28.10.2441

[34] Tesfaye S , Vileikyte L , Rayman G , et al. Painful diabetic peripheral neuropathy: consensus recommendations on diagnosis, assessment and management. Diabetes Metab Res Rev. 2011;27(7):629‐638. 21695762 10.1002/dmrr.1225

[35] Kalyani RR , Tian J , Xue QL , et al. Hyperglycemia and incidence of frailty and lower extremity mobility limitations in older women. J Am Geriatr Soc. 2012;60(9):1701‐1707. 22882211 10.1111/j.1532-5415.2012.04099.x PMC4144067

[36] Blaum CS , Xue QL , Tian J , Semba RD , Fried LP , Walston J . Is hyperglycemia associated with frailty status in older women? J Am Geriatr Soc. 2009;57(5):840‐847. 19484839 10.1111/j.1532-5415.2009.02196.x PMC4120964

[37] Beydoun MA , Popkin BM . The impact of socio‐economic factors on functional status decline among community‐dwelling older adults in China. Soc Sci Med. 2005;60(9):2045‐2057. 15743653 10.1016/j.socscimed.2004.08.063

[38] Drewes YM , den Elzen WP , Mooijaart SP , et al. The effect of cognitive impairment on the predictive value of multimorbidity for the increase in disability in the oldest old: the Leiden 85‐plus Study. Age Ageing. 2011;40(3):352‐357. 21414945 10.1093/ageing/afr010 PMC3080242

[39] Kendig H , Browning C , Pedlow R , Wells Y , Thomas S . Health, social and lifestyle factors in entry to residential aged care: an Australian longitudinal analysis. Age Ageing. 2010;39(3):342‐349. 20233734 10.1093/ageing/afq016

[40] Taş U , Verhagen AP , Bierma‐Zeinstra SM , et al. Incidence and risk factors of disability in the elderly: the Rotterdam Study. Prev Med. 2007;44(3):272‐278. 17184831 10.1016/j.ypmed.2006.11.007

