# Supplementary material for: The pattern of physical disability and determinants of activities of daily living among people with diabetes in Bangladesh
Source: Endocrinol Diabetes Metab. 2022 Aug 14;5(5):e365. doi: 10.1002/edm2.365 (PMC9471590; doi:10.1002/edm2.365)
Supplement: Supplementary file 1 — Appendix S1 [file EDM2-5-e365-s001.docx]

**Supplementary materials:**

**Supplementary Table1: Proportion of lower body disability status among people with diabetes in Bangladesh (n=480)**

| Variable | | Frequency | % |
| --- | --- | --- | --- |
| limitations in walking in a small room | Do the activities without help (no) | 419.0 | 87.3 |
|  | Needful help (yes) | 50.0 | 10.4 |
|  | Unable to do the activities (yes) | 11.0 | 2.3 |
| limitations in bathing | Do the activities without help (no) | 431.0 | 89.8 |
|  | Needful help (yes) | 42.0 | 8.8 |
|  | Unable to do the activities (yes) | 7.0 | 1.5 |
| limitations in transferring from bed to chair | Do the activities without help (no) | 415.0 | 86.5 |
|  | Needful help (yes) | 54.0 | 11.3 |
|  | Unable to do the activities (yes) | 11.0 | 2.3 |
| limitations in using the toilet | Do the activities without help (no) | 394.0 | 82.1 |
|  | Needful help (yes) | 74.0 | 15.4 |
|  | Unable to do the activities (yes) | 12.0 | 2.5 |
| limitations in climbing stairs | Do the activities without help (no) | 319.0 | 66.5 |
|  | Needful help (yes) | 138.0 | 28.8 |
|  | Unable to do the activities (yes) | 23.0 | 4.8 |
| limitations in walking a half mile | Do the activities without help (no) | 365.0 | 76.0 |
|  | Needful help (yes) | 65.0 | 13.5 |
|  | Unable to do the activities (yes) | 50.0 | 10.4 |
| if yes, how long have you had this suffering from this limitation | Below 1 year | 6.0 | 4.5 |
|  | 1 years | 17.0 | 12.9 |
|  | 2 years | 18.0 | 13.6 |
|  | 3 years | 17.0 | 12.9 |
|  | 4 years or more | 74.0 | 56.1 |
| Performance based measure of walking limitations | Able to walk 8 feet at <3 seconds | 10 | 2.1 |
|  | Able to walk 8 feet at 4-5 seconds | 211 | 44.0 |
|  | Able to walk 8 feet at 6-8 seconds | 136 | 28.3 |
|  | Able to walk 8 feet at 8-9 seconds | 92 | 19.2 |
|  | Unable to walk 8 feet at the speed of > 9 seconds | 31 | 6.5 |

Data are expressed as number (percentages). Number of observations across the categories may not add up to the total given number because of missing data.

**Supplementary file 1:** **ADL impairment by Katz activities daily living Scale**

| **Katz Index of Independence in Activities of Daily Living** | | |
| --- | --- | --- |
| **Guideline**  **Activities**  Points (1 or 0) | **Independence**  (1 Point)  **NO** supervision, direction or personal assistance. | **Dependence**  (0 Points)  **WITH** supervision, direction, personal assistance or total care. |
| **BATHING**  Points: __________ | **(1 POINT)** Bathes self completely or needs help in bathing only a single part of the body such as the back, genital area or disabled extremity. | **(0 POINTS)** Need help with bathing more than one part of the body, getting in or out of the tub or shower. Requires total bathing |
| **DRESSING**  Points: __________ | **(1 POINT)** Get clothes from closets and drawers and puts on clothes and outer garments complete with fasteners. May have help tying shoes. | **(0 POINTS)** Needs help with dressing self or needs to be completely dressed. |
| **TOILETING**  Points: __________ | **(1 POINT)** Goes to toilet, gets on and off, arranges clothes, cleans genital area without help. | **(0 POINTS)** Needs help transferring to the toilet, cleaning self or uses bedpan or commode. |
| **TRANSFERRING**  Points: __________ | **(1 POINT)** Moves in and out of bed or chair unassisted. Mechanical transfer aids are acceptable | **(0 POINTS)** Needs help in moving from bed to chair or requires a complete transfer. |
| **CONTINENCE**  Points: __________ | **(1 POINT)** Exercises complete self-control over urination and defecation. | **(0 POINTS)** Is partially or totally incontinent of bowel or bladder |
| **FEEDING**  Points: __________ | **(1 POINT)** Gets food from plate into mouth without help. Preparation of food may be done by another person. | **(0 POINTS)** Needs partial or total help with feeding or requires parenteral feeding. |
| **F1: TOTAL POINTS: ________ SCORING:** 6 = High (*patient independent*) 0 = Low (*patient very dependent)* | | |

If a participant has total score 6: No impairment

If a participant has total score 1-5: has any impairment
